# The Impact of Generic Substitution on Health and Economic Outcomes: A Systematic Review

**DOI:** 10.1007/s40258-014-0147-0

**Published:** 2015-06-20

**Authors:** H. Gothe, I. Schall, K. Saverno, M. Mitrovic, A. Luzak, D. Brixner, U. Siebert

**Affiliations:** Institute of Public Health, Medical Decision Making and Health Technology Assessment, Department of Public Health and Health Technology Assessment, UMIT, University for Health Sciences, Medical Informatics and Technology, Eduard Wallnoefer Center 1, 6060 Hall i.T., Austria; Division of Public Health, Decision Modelling, Health Technology Assessment and Health Economics, ONCOTYROL, Center for Personalized Cancer Medicine Innsbruck, Karl Kapferer Strasse 5, 6020 Innsbruck, Austria; Dresden Medical School “Carl Gustav Carus”, Dresden University of Technology, Fetscherstraße 74, 01307 Dresden, Germany; Department of Pharmacotherapy, University of Utah, 30 S 2000 E, Rm 4410, Salt Lake City, Utah 84112 USA; Center for Health Decision Science, Department of Health Policy and Management, Harvard School of Public Health, 677 Huntington Avenue, Boston, MA 02115 USA; Institute for Technology Assessment, Department of Radiology, Massachusetts General Hospital, Harvard Medical School, 101 Merrimac St., 10th FL, Boston, MA 02114 USA

## Abstract

**Background:**

Generic drugs are considered therapeutically equivalent to their original counterparts and lower in acquisition costs. However, the overall impact of generic substitution (GS) on global clinical and economic outcomes has not been conclusively evaluated.

**Objective:**

To test whether (1) generics and original products yield the same health outcomes, and (2) generic therapies save economic resources versus original therapies.

**Methods:**

We performed a systematic literature review in Medline, Embase, and the Cochrane Database of Systematic Reviews to identify original studies that examine clinical or economic outcomes of GS. After standardized data extraction, reported outcomes were categorized as supporting or rejecting the hypotheses. Each reported outcome was assessed and accounted for supporting and opposing GS. One publication could provide multiple outcome comparisons.

**Results:**

We included 40 studies across ten therapeutic areas. Fourteen studies examined patients on de novo therapy; 24 studies investigated maintenance drug therapy, and two studies considered both settings. Overall, 119 outcome comparisons were examined. Of 97 clinical outcome comparisons, 67 % reported no significant difference between generic drugs and their off-patent counterparts. Of 22 economic comparisons, 64 % suggested that GS increased costs. Consequently, hypothesis (1) was supported but hypothesis (2) was not. We found no major differences among studies that investigated clinical outcomes with de novo or maintenance therapy.

**Conclusion:**

The review suggests that clinical effects are similar after GS. However, economic savings are not guaranteed. More systematic research comparing clinical and economic outcomes with or without GS is needed to inform policy on the use of generic substitution.

## Key Points for Decision Makers

**Table:** 

Studies that analyse the overall clinical and economic consequences of generic substitution in comparison to therapy with originator drugs are lacking.
This review compares clinical outcomes (adherence, adverse events, dose adjustments, concomitant medication, etc.) and economic outcomes (drug costs, outpatient and inpatient services costs, copayments) with or without generic substitution as reported in the literature to assess whether generic substitution leads to the same clinical outcomes while saving healthcare costs in general.
In 67 % of the reported outcome comparisons, clinical effects were similar for generics and their off-patent counterparts.
In 64 % of the reported outcomes, generic substitution was associated with higher costs when compared to therapy with their off-patent counterparts.
Cost savings generated by generic substitution are not guaranteed in the absence of robust research specifically comparing one generic product to another.
The present work includes very heterogeneous studies on different drug types and should be interpreted with caution.

## Introduction

Governments and other healthcare payers are increasingly challenged by rising healthcare expenditures and constrained resources. In countries from the Organization for Economic Co-operation and Development, pharmaceutical expenditures account on average for about 1.5 % of the gross domestic product [1, 2]. Generic substitution (GS) is a commonly employed method for reducing pharmaceutical costs by substituting patented original drugs through generic counterparts with lower acquisition costs [3].

With the passage of the Drug Price Competition and Patent Term Restoration Act (Hatch-Waxman Act) in the USA in 1984, the market entry for generic drugs was streamlined through an abbreviated approval process requiring only a demonstration of bioequivalence for generic approval [4]. Although policies on GS vary from country to country, the policies usually allow the authority to substitute a cheaper generic equivalent for an off-patent original product to a physician (prescribing by international non-proprietary nomenclature) and/or a pharmacist (dispensing of the product preferred by the policy maker or payer).

Such policies are supported by a myriad of studies on GS; most of them were published between the late 1970s and the 1990s when generic substitution was a new and challenging issue [5, 6]. Nevertheless, there is still a lack of appropriate studies involving putatively similar generics—with questionable differences of similarity—which might partly explain the observable variance in clinical responses and side effects. There are several reasons why GS may not be appropriate which are not related to bioequivalence issues [7–9]. For example, inappropriateness is determined by excipient characteristics, but it may also depend on disease entities and clinical conditions, for example, whether a generic drug is applied for de novo or for maintenance therapy.

In order to be considered generic, a drug needs to match the original product in dosage, safety, strength, administration form, quality, performance and intended use. Under these conditions, generics are generally considered to have an equivalent clinical effect when substituted for the original name product [10, 11].

When two generic products are each at the far opposite range of bioequivalence they are equivalent to a brand but not to each other. This results in either over- or under-dosing. Patient confusion and/or nurse confusion in drug intake leads to decreased adherence. Decreased quality of excipients and manufacturing quality can impact drug release and intended action. Any of these scenarios can lead to unintended adverse events that can cost more than the savings in drug costs.

Although bioequivalent generic drugs exist for many original products, it remains controversial whether bioequivalence reflects clinical equivalence. The safety of substituting narrow therapeutic index drugs (NTI), for instance, has been the topic of much debate. Since the therapeutic window of these drugs is relatively small, they can “exhibit limited or erratic absorption, formulation-dependent bioavailability and intra-patient pharmacokinetic variability that requires blood-level monitoring” [12]. Such differences in clinical outcomes can also affect the intended economic savings. If, for instance, rates for adverse events were higher in patients switching to generic drugs, overall expenses may be higher than expected or may even exceed the amount spent previously for the original drug.

Considering the variety of drugs and drug types, literature pertaining to clinical and economic outcomes of GS may be more robust in some therapeutic areas or treatment stages than others. However, to date, we are not aware of any research that has attempted to summarize the entire body of evidence on the impact of GS across multiple therapeutic areas including clinical and economic outcomes.

Therefore, the aim of this study was to evaluate whether (1) original medications and their corresponding generic equivalents yield the same health outcomes and (2) whether generic therapies save economic resources in contrast to original therapies when evaluating health outcomes and economic outcomes. A systematic review of the published literature was conducted for patients starting a new therapy (de novo) and patients on maintenance therapy.

## Methods

### Literature Search

A systematic literature search was performed in the following electronic databases: Medline using the PubMed interface, the Cochrane Database of Systematic Reviews, and Embase. A comprehensive search syntax (see “Appendix”) that included the terms “generic substitution”, “drug substitution”, “drug switching”, “adverse event”, “drug safety”, “risk benefit ratio”, “cost containment”, “health economic”, “adherence”, “compliance”, “persistence” and “medication adherence” was run through PubMed and the Cochrane Database of Systematic Reviews in September 2012. A subsequent literature search was performed in November 2012 using a less distinct syntax with the term “generic substitution” for titles and abstracts in Medline, Cochrane Database of Systematic Reviews and Embase. All searches were limited to the publication years from 2000 to 2012. In addition, cited references of the included studies were searched manually.

### Literature Selection

We selected only full publications of original research studies that focused on GS, examined clinical or economic outcomes and included either a separate or pre-post comparator group. Exclusion criteria were: (a) publication language was neither English nor German, (b) study design implied no original research, (c) study endpoints did not include clinical or economic outcomes, (d) study intervention was not limited to generic switching but comprised broader options such as therapeutic interchange. Additionally, studies were excluded that exclusively assessed stakeholder opinions, satisfaction or knowledge of GS. Likewise, budget impact analyses, which projected cost or market consequences due to generic product entry to the marketplace, were excluded. Both selection and data extraction were realized by two independent scientists, who were supplemented by a third scientist or discussion in cases of disagreement.

### Data Extraction

We developed a standardized assessment form, containing the following domains: citation, funding source/conflict of interest, research question/objective, specific drug, NTI drug (yes/no), drug class, reference comparator, results, outcome types, adherence measures, conclusions, study type and limitations reported by the authors. For clinical outcomes, we extracted reported endpoints on dose adjustment, additional medication, adherence, adverse events, healthcare utilization, surrogate outcome parameters, and others. For economic outcomes, we extracted reported endpoints on drug costs of the investigated drug as well as additional drugs, outpatient and inpatient healthcare utilization costs, co-payments and healthcare costs in general.

De novo and maintenance therapy regimens were defined as whether or not patients had received the investigated active ingredient before study commencement. Consequently, patients who were initiated on a chronic treatment were classified as receiving de novo therapy.

### Data Synthesis

We condensed the study data on each outcome by classifying them as either supporting or opposing each of the two hypotheses. According to the definition of generic drugs, clinical outcomes were categorized as supporting the first hypothesis if no statistically significant difference was found between original and generic drug therapy or in case clinical outcomes yielded statistically significant better outcomes, e.g., lower adverse events or higher adherence rates, than original drugs. The second hypothesis was considered supported if the therapy costs under GS were significantly reduced. Finally, each outcome comparison was counted as supporting or not supporting and the percentage of supporting evidence of the total number of comparisons was derived for each hypothesis.

As studies often examined several outcome measures, the sum of outcome comparisons differs from the number of studies included. Therefore, we distinguished between terminology of “studies” and “outcome comparisons”.

## Results

### Systematic Literature Search and Study Characteristics

**Fig. 1:**
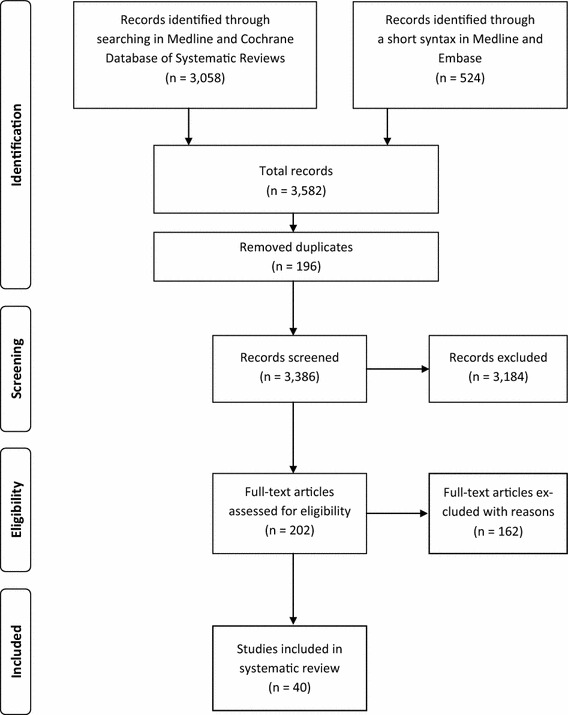
PRISMA statement. The flow diagram depicts the flow of information through the different phases (identification–screening–eligibility—included) of the systematic review. It maps out the number of records in each phase and shows how many studies have been included or excluded, respectively

After removing duplicates, the systematic literature search yielded 3,386 citations. After title and abstract screening, 202 publications remained in the database. By means of full text examination, 162 studies were excluded, most of them (*n* = 68) for formal criteria, that is, the publication type turned out to be a comment, editorial, letter to the editor or an extended abstract. Forty-three studies were excluded because they did not focus on economic, clinical, and/or humanistic outcomes. Thirty-five publications were excluded due to study type (review, case-studies, hypothetical cost-saving analyses). Ten studies were excluded because they focused on therapeutic interchange rather than generic substitution, and six articles were excluded because instead of GS the main interventions were policy changes or price adjustment measures. In the end, 40 studies (publications [20–59] in the reference list) matched the selection criteria and were included in the review (Fig. 1).

Included studies were performed in 16 countries from three continents (20 studies from North America, eight studies from Europe and 12 studies from Asia). While 80 % of the studies investigated clinical outcomes only, 7.5 % investigated economic outcomes only and 12.5 % examined both clinical and economic outcomes. The studies covered ten therapeutic classes, four of which included NTI drugs (13 studies on NTI drugs of 40 included studies = 32 %).

The selected studies were largely heterogeneous regarding therapeutic categories, types of therapy (i.e., chronic/ maintenance therapy vs. new/de novo therapy), study design, switching sequences (e.g., parallel patient groups vs. cross-over designs), settings, funding and others. (Tables 1, 2) Study designs included randomized controlled trials (17.5 %), prospective cohort studies (10 %) and retrospective cohort studies (60 %). Additionally, there was one decision model, one non-randomized controlled trial (2.5 % respectively) and three studies with study designs that remained unclear (7.5 %). The time frame of the studies ranged from eight weeks for interventional studies to 93 months in observational studies. Similarly, population size ranged from eight patients to 221,881 patients (Table 1).

**Table 1:** Overview of studies included in the review

Study no.	References	Therapeutic category	De novo/maintenance therapy	Outcome type	Study population (*n*)
20.	Alessi-Severini et al. [20]	Antipsychotics	*M*	*C*	58
21.	Amit et al. [21]	Antiarrhythmics	*M*	*C*	114
22.	Andermann et al. [22]	AED	*M*	*C*	1,354
23.	Araszkiewicz et al. [23]	Antipsychotics	*D*	*C*	85
24.	Assawawitoontip and Wiwanitkit [24]	Antihypercholesterolemics (statins)	*D*	*C*	48
25.	Boh et al. [25]	Antihypercholesterolemics (statins)	*D*	*C*	138
26.	Burkhardt et al. [26]	AED	*M*	*C*	8
27.	Carius and Schulze-Bonhage [27]	AED	*M*	*C*	39
28.	Chaluvadi et al. [28]	AED	*M*	*C*	245
29.	Diarra et al. [29]	Immunosuppressives	*M*	*C*	59
30.	Duh et al. [30]	AED	*M*	*E*	1,142
31.	Duh et al. [31]	AED	*M*	*C + E*	948
32.	Fujii et al. [32]	Oncology medication (folic acid)	*D*	*C*	42
33.	Ghate et al. [33]	Anticoagulants	*D*	*C*	37,756
34.	Halkin et al. [34]	Anticoagulants	*M*	*C*	975
35.	Halkin et al. [35]	Osteoporosis (bisphosphonates)	*D*	*C*	6,962
36.	Haroldson et al. [36]	Immunosuppressives	*M*	*C* + *E*	30
37.	Hartung et al. [37]	AED	*M*	C	616
38.	Helderman et al. [38]	Immunosuppressives	*D*	*C* + *E*	227
39.	Jeong et al. [39]	Anticoagulants	*M*	*C*	20
40.	Kim et al. [40]	Antihypercholesterolemics (statins)	*D*	*C*	211
41.	Kluznik et al. [41]	Antipsychotics	*M*	*C*	49
42.	Labiner et al. [42]	AED	*D* + *M*	*C*	33,625
43.	Lai et al. [43]	Osteoporosis (bisphosphonates)	*D* + *M*	*C*	131
44.	Layton and Barbeau [44]	Antipsychotics	*M*	*E*	100^a^
45.	Lee et al. [45]	Anticoagulants	*M*	*C*	35
46.	LeLorier et al. [46]	AED	*M*	*E*	671
47.	LeLorier et al. [47]	AED	*M*	*C*	671
48.	McDevitt-Potter et al. [48]	Immunosuppressives	*M*	*C* + *E*	70
49.	Milligan et al. [49]	Anticoagulants	*M*	*C*	182
50.	Momper et al. [50]	Immunosuppressives	*M*	*C*	103
51.	Narayanaswamy et al. [51]	Glaucoma medication (prostaglandin analogue)	*D*	*C*	30
52.	Pamugas et al. [52]	Immunosuppressives	*D*	*C*	60
53.	Ringe and Moller [53]	Osteoporosis (bisphosphonates)	*D*	*C*	186
54.	Sajbel et al. [54]	Antipsychotics	*M*	*C*	17
55.	Ström and Landfeldt [55]	Osteoporosis (bisphosphonates)	*D*	*C*	36,433
56.	Ude et al. [56]	Antihypertensives	*M*	*C*	221,881
57.	Van Wijk et al. [57]	Antihypertensives	*D*	*C*	1,028
58.	Witt et al. [58]	Anticoagulants	*M*	*C* + *E*	2,299
59.	Wiwanitkit et al. [59]	Antihypercholesterolemics (statins)	*D*	*C*	43

*D* de novo therapy, *M* maintenance therapy, *D* *+* *M* both, *C* clinical, *E* economic, *C* *+* *E* clinical and economic, *AED* antiepileptic drug

^a^Patient number in the health economic decision model

**Table 2:** Study characteristics

Characteristic	De novo therapy	Maintenance therapy	Both
	*n* = 14 studies	*n* = 24 studies	*n* = 2 studies
Outcome type			
Clinical only	13	17	2
Economic only	–	3	–
Clinical and economic	1	4	–
Country			
USA	2	11	1
Canada	–	6	–
Asia (Israel, Thailand, Japan, Taiwan, India, Philippines, Malaysia)	7	4	1
Europe (Germany, Austria, Netherlands, Poland, Slovenia, Sweden)	5	3	–
Funding			
Industry (pharmaceutical and other)	6	12	1
Academia / healthcare organizations / public	1	4	–
No funding	2	–	1
Not stated	5	8	–
Therapeutic category			
Antiepileptics	–	9	1
Antiarrhythmics	–	1	–
Anticoagulants	1	5	–
Antihypercholesterolemics	4	–	–
Antihypertensives	1	1	–
Antipsychotics	1	4	–
Ocular (glaucoma)	1	–	–
Immunosuppressives	2	4	–
Oncology	1	–	–
Osteoporosis	3	–	1
Narrow therapeutic index (NTI)	2	10	1
Study design			
Interventional studies	5	4	–
RCT, cross-over (brand to generic and v.v.)	3	2	–
RCT, parallel groups, no switch (brand only vs. generic only)	2	–	–
Controlled trial, non-randomized	–	1	–
Simple substitution (brand to generic)	–	1	–
Observational studies, prospective	1	2	1
Parallel groups, no switch (brand only vs. generic only)	1	–	–
Simple substitution (brand to generic)	–	2	–
Simple substitution (brand to generic vs. brand or generic only)	–	–	1
Observational studies, retrospective	7	16	1
Parallel groups, no switch (brand only vs. generic only)	3	1	–
Simple substitution (brand to generic, incl. switch-back if appl.)	–	12	–
Simple substitution (brand to generic vs. brand or generic only)	3	–	–
Cross-over (brand to generic and v.v.)	–	1	–
Open cohort (all possible switches)	1	2	1
Decision analytic model	–	1	–
Simple substitution (brand to generic vs. brand or generic only)	–	1	–
Unclear	1	2	–
Simple substitution (brand to generic)	–	2	–
Parallel groups, no switch (brand only vs. generic only)	1	–	–

We identified 14 studies with a study population receiving de novo therapy and 24 studies on maintenance therapy. Two studies examined both de novo and maintenance therapy under generic substitution. Study characteristics grouped by de novo or maintenance therapy remained heterogeneous. However, studies on economic outcomes seemed to examine maintenance therapy more often (seven of eight economic outcomes comparison studies) than de novo therapy (one of eight studies). In addition, GS of NTI drugs was examined more often in maintenance therapy (10 of 12 NTI studies), than in de novo therapy (2 of 12 NTI studies). Accordingly, maintenance therapy studies covered more sensitive therapeutic categories than studies on de novo therapy. While studies on maintenance therapy included mostly antiepileptic drugs (AEDs), anticoagulants, antipsychotic drugs and immunosuppressive drugs, studies on de novo therapy included mainly antihypercholesterolaemic drugs or osteoporosis medication and others.

### Clinical Outcomes

Clinical outcomes were examined by 37 of 40 studies that led to 97 comparisons of clinical outcomes. These studies covered all ten therapeutic classes as described above and investigated about 26 different drugs. Table 3 displays an overview of our findings sorted by de novo and maintenance therapy.

**Table 3:** Clinical outcome comparisons (study references in square brackets)

Clinical outcomes	De novo patients starting on generics		Maintenance patients switching to generics		Both: de novo and maintenance patients		Total *n* = 37
	*n* = 14 studies		*n* = 21 studies		*n* = 2 studies		
	Evidence supporting generic use	Evidence opposing generic use	Evidence supporting generic use	Evidence opposing generic use	Evidence supporting generic use	Evidence opposing generic use	
Dose adjustments	3	–	7	7	–	–	17
	[23, 25, 52]		[20, 21, 26, 29, 49, 50, 54]	[22, 30, 35, 37, 47, 48, 58]			
Concomitant medication	3	–	3	4	–	1	11
	[32, 34, 55]		[21, 26, 37]	[22, 31, 47, 56]		[42]	
Adherence	1	3	3	–	1	–	8
	[57]	[34, 53, 55]	[39, 45, 46]		[43]		
Adverse events	8	3	11	3	1	–	26
	[23–25, 32, 40, 51, 52, 59]	[33, 38, 53]	[20, 21,29, 36, 39, 45, 48–50, 54, 58]	[27, 28, 41]	[43]		
Healthcare utilization	4	–	5	2	–	1	12
	[23, 34, 38, 57]		[20, 21, 35–37]	[31, 47]		[42]	
Surrogate endpoints	6	2	9	5	–	–	22
	[24, 25, 32, 40, 52, 59]	[51, 53]	[20, 29, 36, 39, 45, 48, 49, 54, 58]	[26, 27, 35, 41, 50]			
Others	–	–	–	–	–	1	1
						[43]	
Overall equivalence	25	8	38	21	2	3	97

Of the investigated clinical outcome comparisons in de novo therapy, 76 % found generic drugs to produce similar clinical outcomes when compared with the original reference drugs (25 of 33 clinical outcome comparisons). Likewise, 64 % of the examined clinical outcome comparisons demonstrated equal effectiveness with GS (38 of 59 clinical outcome comparisons) in maintenance therapy patients. Of the studies that included both therapy types (treatment stages), 40 % reported similar clinical outcomes with generic substitution.

All in all, 67 % of the clinical outcome comparisons included in this analysis revealed no difference between original and generic drug therapy effectiveness with generic therapy, and therefore, supported our first hypothesis.

### Economic Outcomes

Regarding economic outcomes, we extracted reported data on drug costs for the investigated drug and/or potential co-medication, as well as costs on healthcare utilization (inpatient and outpatient) and co-payments.

Of the 40 included studies, three examined economic outcomes only whereas another five studies examined both economic and clinical outcomes. These eight studies led to 22 outcome comparisons. The most frequent study design underlying the economic analyses was retrospective database analysis (five of eight economic studies). Another study projected actual economic observations from a Canadian retrospective open cohort study to a US setting using mathematical approaches, while another study determined the relapse incidence at which switching to a generic drug was cost-neutral through a simple decision analytical model. Only one prospective cohort study was found within this economic context.

These eight studies examined economic outcomes of GS in epilepsy patients treated with AEDs (37.5 %), organ transplant recipients treated with immunosuppressives (37.5 %), atypical neuroleptics (12.5 %) and anticoagulants in patients with continuous use of warfarin (12.5 %).

Seven of eight economic studies (87.5 %) found drug acquisition costs of the investigated medication to be lower for generic drugs (Table 4). Five studies identified drug costs for additional medication during generic drug use. In four of them (80 %) the supplementary medication costs exceeded the cost savings obtained from lower costs for the investigated medication due to GS. Total costs for inpatient and outpatient healthcare utilization (e.g., physician visits, hospitalization visits, etc.) were always lower with use of original products. Only costs that arose during the study periods ranging from 180 days to 5 years were included.

**Table 4:** Economic outcome comparisons (study references in square brackets)

Economic outcomes	De novo patients starting on generics		Maintenance patients switching to generics		Total *n* = 8
	*n* = 1 study		*n* = 7 studies		
	Evidence supporting generic use	Evidence opposing generic use	Evidence supporting generic use	Evidence opposing generic use	
Drug costs of investigated drug	–	1	7	–	8
		[38]	[30, 31, 36, 44, 46, 48, 58]		
Drug costs of concomitant medication	–	1	–	4	5
		[38]		[30, 31, 46, 58]	
Outpatient services costs	–	1	–	3	4
		[38]			
				[31, 46, 58]	
Inpatient services costs	–	1	–	3	4
		[38]		[31, 44, 46]	
Co-payments	–	–	1	–	1
			[48]		
Overall evidence—economic outcomes	–	4	8	10	22

In the consolidated evaluation of economic outcome comparisons, 64 % indicated that staying with an original product incurs lower costs than GS. One of these studies examined economic outcomes for de novo therapy, in this case immunosuppressives after organ transplantation, and found total costs to be lower with originator therapy. In the group of maintenance therapy, 55 % of the economic comparisons opposed our second hypothesis.

Seven of eight economic studies calculated the total healthcare costs according to their reported economic outcomes. Contrary to our finding from counting whether or not outcome comparisons supported our second hypothesis, half of these studies found that GS leads to lower costs.

## Discussion

Our findings suggest that 67 % of the evidence reported clinical similarity of GS as compared to original drug therapy, whereas 64 % of the comparisons of economic outcomes suggest costs to be lower when using original drugs. Accordingly, our first hypothesis was supported and our second hypothesis was rejected.

When stratifying the groups by de novo and maintenance therapy, we found a slight difference among studies on clinical outcomes: 76 % of clinical comparisons found similar effects for generics in de novo therapy and 64 % of clinical comparisons found similar effects in generics in maintenance therapy. Likewise, all economic comparisons in patients receiving de novo therapy (one study) versus 56 % in maintenance therapy (seven studies) revealed lower cost with originator therapy. None of the economic outcome comparisons revealed similar costs. However, the low number of studies in each group limits the generalizability of these results.

The majority of economic studies are related to sensitive therapeutic categories and maintenance therapy. Data on economic consequences of generic drug substitution in less sensitive therapeutic categories such as antihypercholesterolemics or osteoporosis were missing or did not meet our inclusion criteria. Thus, our review may suffer from publication bias and the economic conclusions are only relevant to these sensitive therapeutic areas. In the absence of evidence, no conclusions on economic advantages of one policy (e.g., generic substitution) over the other (e.g., originator therapy) can be drawn for less sensitive therapeutic areas. Moreover, with only one economic study analysing the economic impact of generic substitution for de novo therapy, the result must not be generalized. Hence, more evidence is needed to examine the economic impact of generic substitution in a real-life therapy situation, where chronic generic therapy may involve multiple substitution and thus multiple switching. Total healthcare costs were calculated in seven of eight studies. Half of these found cost reductions to be realized with generic drugs, whereas the other half found costs to be lower with the original drug. In contrast, our dichotomous classification supporting or opposing GS resulted in a stronger preference for originator drugs (lower healthcare cost in general).

The high heterogeneity among the cost types reported in the economic studies is also important to note, even among those which claimed to take the payer perspective. If only drug costs of the investigated drug and concomitant medication were examined, findings were likely to show cost reductions after GS. However, if dose adjustments, co-medication or healthcare utilization were considered depending on the rates of adverse events, GS no longer realized cost reductions. Instead, economic outcomes became more preferable in original drugs. It may be speculated that such results depend on additional factors such as therapeutic area, patient age or education level, number of medications or general healthcare context.

These considerations also apply to our analysis on clinical outcomes. If we focus on patient-relevant outcomes such as additional medication, adherence and adverse events only, disregarding surrogate outcomes or others, 67 % (12 out of 18 clinical outcome parameters for these three dimensions) of the categorized data on clinical outcomes remain in favour of our hypothesis of similar clinical effects. Evidence on all clinical outcomes did support the first hypothesis more often in de novo therapy (76 % of the relevant 33 clinical outcome comparisons) than in maintenance therapy (64 % of the relevant 59 clinical outcome comparisons).

This review has multiple limitations which should be considered when interpreting the results. We used a dichotomous classification of evidence, whether studies reported a statistically significant difference between generic and original drugs or not. We used this simplifying approach as the broad topic of this review resulted in a very heterogeneous pool of studies which impaired comparability and information had to be condensed in order to be able to provide a meaningful summary on the topic. Moreover, studies were not critically appraised regarding methodological aspects in a formal manner. For example, a comparison of therapy with one generic drug versus therapy with the originator drug in an 8-week randomized clinical study may answer a different question to a 2-year observational study in a real-life setting including multiple switching under a generic substitution policy. Among the included studies, three compared original drugs with their generic counterpart as well as drugs of different active ingredients [13–15]. Of these only the data comparing generics with the original reference drug were included. Additionally, our analysis did not include substitution between biological originator drugs and biosimilars. Finally, we took a semi-quantitative approach to summarize the evidence. Rather than pooling results in a formal meta-analysis, we simply counted outcome comparisons in favour of or against the compared strategies, and therefore did not weigh studies by sample size or precision. We also ignored dependency between multiple comparisons within one study and did not perform formal statistical tests or uncertainty analysis. Therefore, our analyses are merely exploratory, and should be interpreted with appropriate caution.

Of the included studies, 22 displayed a direct financial interest in their investigated drugs. In 55 % of these studies, the potential interest was linked to the original product, in 31 % of the studies this was linked to a generic and three studies (14 %) could not be specified, for instance due to a variety of author honoraria from several industry partners. Among those with a link to an original product, ten of twelve studies (83 %) found original drugs to generate preferable outcomes (clinical or economic), and of the studies with a link to a generic product, six of seven studies (86 %) found generics to be the dominant drug. Therefore, detection bias should be further explored in subsequent analyses.

We found economic outcomes to be more favourable in original drugs than in generics, which is in accordance with Duh et al. [16] who detected higher than expected healthcare costs in generic AEDs and revealed that GS may even increase healthcare costs. The included studies of this review mainly suggested further underpinnings of their specific findings, or extensions of the covered methodology such as reporting of additional outcomes, broader or different populations or different medications. Considering the variety of original research that we identified with our systematic literature search, few systematic reviews or meta-analyses have been published focusing only on specific aspects of GS, mainly within sensitive therapeutic classes [16–19]. Kesselheim et al. [18], for instance, investigated the impact of generic AEDs on clinical outcomes, namely seizure control, and hypothesized superiority of original products. Although the meta-analysis showed “no difference in the odds of uncontrolled seizure for patients on generic medications compared with patients on original-name medications”, observational studies found health services utilization to be slightly increased after GS. The authors attributed this insight to a detection bias caused by “concern from patients or physicians about the effectiveness of generic AEDs”. Similarly, economic outcomes of AEDs were assessed by Duh et al. [16] who found generic AEDs to cause higher healthcare costs than their original counterparts in both stable and unstable epilepsy patients. Multiple-generic substitution increased this effect even more.[ The findings of these two reviews apply to our results in regard to both clinical and economic outcomes. Desmarais et al. [17] investigated the clinical equivalence of original and generic psychotropic medications such as anticonvulsants and mood stabilizers. Besides raising compliance issues, generics caused clinical deterioration, adverse effects and changes in pharmacokinetics while leading to lower than expected cost savings. The authors therefore suggested generic switching of psychotropic medication to be advised only on an individual basis while simultaneously monitoring the switch [17].

Another systematic review by Kesselheim et al. [18] focused on clinical evidence in cardiovascular disease while also examining related opinions of editorialists. The analyses did not reveal superiority of either drug type, but found a considerable amount of editorials opposing generic drugs. While these reviews addressed mostly sensitive therapeutic categories, most of them found generics to result in similar clinical effects. In accordance with our findings, these reviews found economic consequences to be higher in periods of generic use compared to periods of original use. Altogether, these reviews revealed reservations and concerns in treatment routines of generics.

Based on our findings, we are able to observe suggested trends based on a significantly heterogeneous review. Future work will look to validate these trends with more robust methods as demonstrated in other more homogenous clinical and economic reviews.

We detected a need for more systematic reviews that determine the impact of GS on clinical and economic outcomes while differentiating between therapeutic classes since substitution may be more sensitive in some areas than others due to their pharmaceutical characteristics. Moreover, patient groups should be subdivided into patients receiving a new and those receiving a maintenance therapy as generics may have different effects for those who start a new treatment than for those who are already stable on a treatment. This is especially true for sensitive therapeutic classes not only with a narrow therapeutic window (e.g., AEDs), but also others such as drugs used in schizophrenia.

## Conclusion

Despite mainly similar clinical effects, our analyses suggest that original to generic drug substitution may not reduce costs, particularly in sensitive therapeutic areas such as with AEDs or immunosuppressive drugs. Evidence on clinical outcomes was slightly more distinct in studies with patients on maintenance therapy than in treatment-naïve patients. As we found only one economic study in patients receiving de novo therapy, a comparison with the remaining seven studies with maintenance therapy patients seems unreasonable. Since evidence is heterogeneous and in this context influenced by several other influencing factors, further research is needed in GS. Studies should ideally be based on real world evidence to determine the true comparative effectiveness of GS. Preference should be given to interventional approaches which randomize patients to generic and brand arms, control for co-morbidities, disease severity, maintenance versus de novo therapy, etc. We therefore recommend that research in GS focuses on systematic approaches in therapeutic areas in order to analyse outcomes and uncertainty.

## Appendix: search strategy

### Detailed search syntax for Medline via PubMed and the Cochrane Database of Systematic Reviews

**Table:**

Search	Query
1	“Drug substitution” [mesh]
2	“Drugs, generic” [mesh]
3	“Drug substitutions” or “substitutions, drug” or “drug switching” or “switching, drug” or “therapeutic substitution” or “generic substitution” or “generic substitutions” or “generic drug”
4	“Generic medicine” or “generic product” or originator or “branded generic” or “mature products”
5	1 or 2 or 3 or 4
6	“Drug toxicity” [mesh]
7	“Adverse event” or “adverse events” or “adverse effect” or “treatment failure” or “drug safety” or “drug interaction” or “patient safety” or “clinical response” or “side effects” or “risk-benefit balance” or “risk benefit balance” or “risk-benefit ratio” or “risk benefit ratio” or “adverse outcomes”
8	“Cost containment” or “cost saving” or “cost savings” or “cost minimization” or “health economic” or “resource use” or “resource utilization”
9	Adherence or compliance or persistence or “medication adherence”
10	6 or 7 or 8 or 9
11	5 and 10 limits: (language: eng, ger; published: after 2000)
	The query in Medline via Pubmed resulted in 3,030 hits
	The query in the Cochrane Database of Systematic Reviews resulted in 28 hits
